# Titanium Dioxide Nanoparticle Humidity Microsensors Integrated with Circuitry on-a-Chip

**DOI:** 10.3390/s140304177

**Published:** 2014-03-03

**Authors:** Yu-Chih Hu, Ching-Liang Dai, Cheng-Chih Hsu

**Affiliations:** 1 Department of Mechanical Engineering, National Chung Hsing University, Taichung 402, Taiwan; E-Mail: willy06101024@yahoo.com.tw; 2 Department of Electro-Optical Engineering, Yuan Ze University, Taoyuan 320, Taiwan; E-Mail: cchsu@saturn.yzu.edu.tw

**Keywords:** integrtaed humidity microsensor, titanium dioxide, readout circuit

## Abstract

A humidity microsensor integrated with a readout circuit on-a-chip fabricated using the commercial 0.18 μm CMOS (complementary metal oxide semiconductor) process was presented. The integrated sensor chip consists of a humidity sensor and a readout circuit. The humidity sensor is composed of a sensitive film and interdigitated electrodes. The sensitive film is titanium dioxide prepared by the sol-gel method. The titanium dioxide is coated on the interdigitated electrodes. The humidity sensor requires a post-process to remove the sacrificial layer and to coat the titanium dioxide. The resistance of the sensor changes as the sensitive film absorbs or desorbs vapor. The readout circuit is employed to convert the resistance variation of the sensor into the output voltage. The experimental results show that the integrated humidity sensor has a sensitivity of 4.5 mV/RH% (relative humidity) at room temperature.

## 1. Introduction

Humidity sensors are important devices for application in industrial and electronic equipment. Various traditional sensors have been miniaturized as microsensors using micromachining technology. Micromachined sensors have the benefits of high performance, low cost, small size, and easy mass-production [1]. Many humidity microsensors have been manufactured using micromachining technology. For instance, Chen *et al.* [2] used micromachining technology to fabricate a capacitive relative humidity sensor in which its sensitive material was zinc oxide. The humidity-sensitive ZnO nanorods were synthesized by the thermal decomposition, and then deposited on the micromachined electrodes by dielectrophoretic manipulation. Kim *et al.* [3] developed a capacitive humidity sensor using microelectromechanical system (MEMS) technology. The structure of the humidity sensor was composed of interdigitated electrodes and a polyimide sensing layer. The height of interdigitated electrodes was increased in order to enhance the capacitance and sensitivity of the sensor, and it had a sensitivity of 37.1 fF/RH% (relative humidity). Lee *et al.* [4] presented an integrated humidity sensor with micropumps. The valve-less micropumps in the sensor system were fabricated using micromachining process of a deep reactive ion etching and an anodic bonding step. The maximum flow rate of the micropumps was 0.176 μL/min. The sensitivity of the humidity sensor with pumping was 10 times higher than it was without pumping. Lee *et al.* [5] manufactured a novel high-speed polyimide-based humidity sensor using a combination of isotropic and anisotropic etching steps with inductively coupled plasma and a localized curing of polyimide film on microhotplate. The polyimide capacitive humidity sensor showed a sensitivity of 0.77 pF/RH%. Chen *et al.* [6] proposed a humidity microsensor with a micromachined silicon dioxide cantilever beam. The fabrication of the sensor combined the isotropic and anisotropic dry etching of inductively couple plasma to release the silicon dioxide cantilever beam. Deflection amplitude of the silicon dioxide microcantilever beam was observed upon exposure to 1% relative humidity. Su *et al.* [7] employed MEMS technology and thick-film technology to make a resistive humidity microsensor. The microsensor consisted of a suspended planar membrane, metal electrodes and a humidity-sensing film. Metal electrodes were located on the surface of the membrane, and the humidity-sensing film of poly-[3-(methacryloylamino)propyl] trimethylammonium chloride and SiO_2_ was coated on the top of the electrodes. The conductivity of the humidity-sensing film changed upon adsorption/desorption of water vapor. Lazarus and Fedder [8] fabricated a capacitive humidity microsensor using complementary metal oxide semiconductor (CMOS)-MEMS technique. The fabrication of the microsensor included adding oxide pillars to hold the plate apart, spin coating polymer to the electrodes, adding a polysilicon heater and etching away excess polymer in the release holes. The humidity sensor had a sensitivity of 0.21% change in capacitance per RH%. A capacitive humidity microsensor, proposed by Zhao *et al.* [9], was fabricated using the CMOS process. The microsensor was composed of a polysilicon heater, aluminum interdigitated electrodes and a humidity-sensing film of polyimide. Both the hysteresis and the recovery time of the sensor were improved based on the polysilicon heater. Yang *et al.* [10] utilized the commercial CMOS process to produce a resistive humidity microsensor. The sensitive film of the sensor was polyaniline doping polyvinyl alcohol (PVA) prepared by sol-gel method. The sensitivity of the humidity sensor was 12.6 kΩ/RH%. The humidity microsensors [2–10] were not integrated with circuitry on-a-chip. Microsensors integrated with circuitry on-a-chip have the benefits of low package cost and high performance [1]. In this work, we manufacture a humidity sensor integrated with a readout circuit on chip.

The commercial CMOS process has been used to develop various microsensors and microactuators [11–14]. Microsensors fabricated by this process can be integrated with circuitry on-a-chip [15–18]. In this study, a humidity sensor with a readout circuit on chip is manufactured using the CMOS process. Titanium dioxide prepared by the sol-gel method is adopted as the sensitive material of the sensor. The sensor needs a post-process [19–21] to coat the sensitive material. The post-process contains etching the sacrificial oxide layer and depositing the sensitive film.

## 2. Structure of the Humidity Sensor

**Figure 1.**
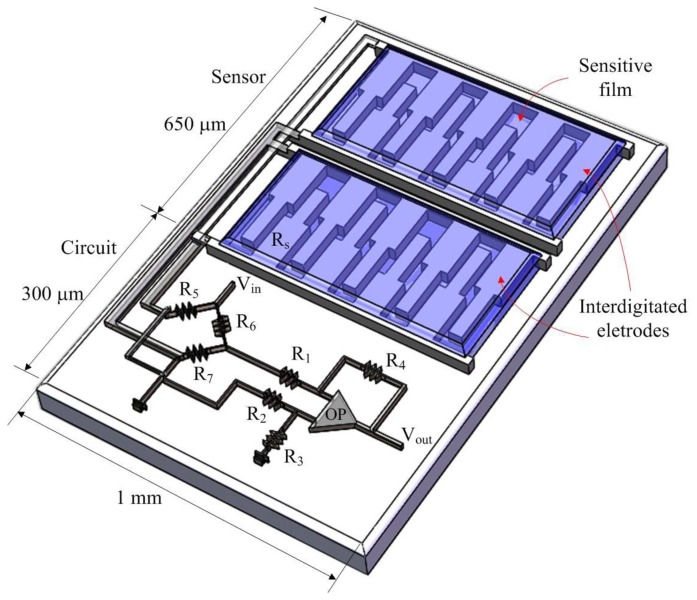
Schematic structure of the integrated humidity sensor.

Figure 1 illustrates the schematic structure of the integrated humidity sensor chip. The integrated microsensor chip consists of a humidity sensor and a readout circuit. The humidity sensor is composed of interdigitated electrodes and a sensitive film. The interdigitated electrodes are 300 μm long and 6 μm thick, and their widths are both 5 and 10 μm. The gap between the interdigitated electrodes is 10 μm. Material of the sensitive film is titanium dioxide, and the sensitive film is coated on the interdigitated electrodes. When the sensitive film of titanium dioxide adsorbs vapor, the conductivity of the film increases because the electrons in the film increases, leading to the resistance of the film decreases [22,23]. Thereby, the humidity sensor is a resistive type. The humidity sensor produces a change in resistance when the sensitive film adsorbs or desorbs vapor.

As shown in Figure 1, the integrated microsensor contains a readout circuit [24] to convert the resistance variation of the humidity sensor into the output voltage, where *OP* represents the operational amplifier, *V*_in_ is the input voltage of the circuit; *V*_out_ is the output voltage of the circuit; *R*_s_ is the resistance of the humidity sensor; *R*_1_–*R*_7_ are the resistances of the circuit.

**Figure 2.**
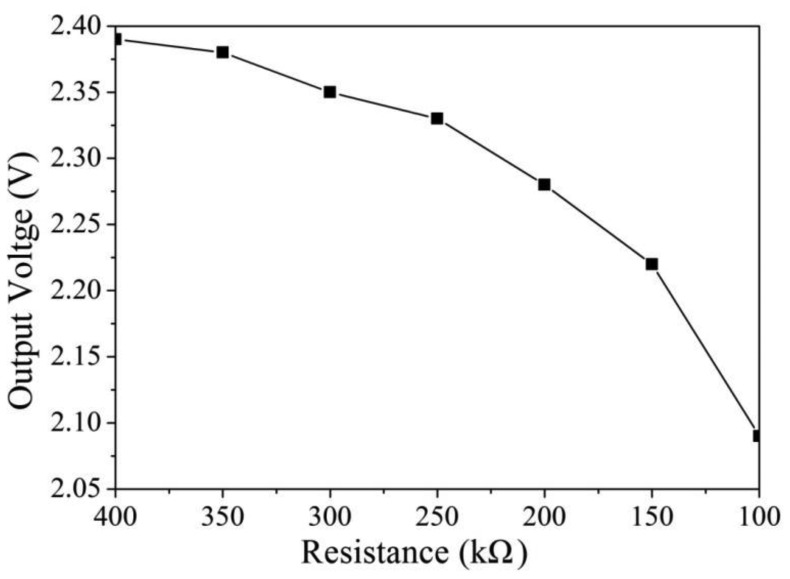
Simulated results of the output voltage for the readout circuit.

**Figure 3.**
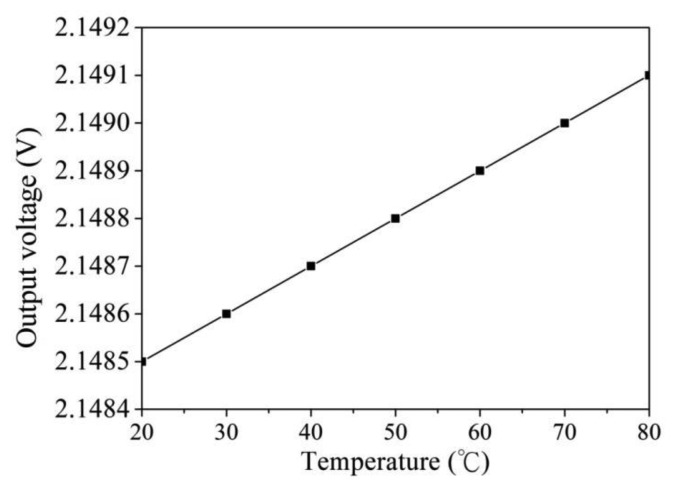
Relation between output voltage and temperature for the circuit.

The software HSPICE (Synopsys Taiwan Co., Ltd., Hsinchu, Taiwan) is utilized to simulate the characteristics of the readout circuit. Figure 2 shows the simulated results of output voltage for the readout circuit. In this simulation, the resistances *R*_1_ = 5 kΩ, *R*_2_ = 5 kΩ, *R*_3_ = 30 kΩ, *R*_4_ = 30 kΩ, *R*_5_ = 20 kΩ, *R*_6_ = 30 kΩ, *R*_7_ = 10 kΩ and *R*_s_ = 100 kΩ are set, and the input voltage *V*_in_ is 2.5 V. The bias voltage of the amplifier is 3.3 V. The resistance of the humidity sensor varies from 400 to 100 kΩ. The simulated results revealed that the output voltage of the readout circuit decreased from 2.39 to 2.14 V as the resistance of the sensor changed from 400 to 100 kΩ. To characterize the relation between the output voltage and temperature for the readout circuit, the readout circuit is simulated at different temperatures. Figure 3 shows the simulated results of output voltage for the readout circuit at different temperatures. In this investigation, the bias voltage of the amplifier is 3.3 V, and the input voltage *V*_in_ is 2.5 V. The resistance of the humidity sensor is 120 kΩ, and the resistances are *R*_1_ = 5 kΩ, *R*_2_ = 5 kΩ, *R*_3_ = 30 kΩ, *R*_4_ = 30 kΩ, *R*_5_ = 20 kΩ, *R*_6_ = 30 kΩ and *R*_7_ = 10 kΩ. The temperature changes from 20 to 80 °C. The results showed that the output voltage of the readout circuit increased from 2.1485 to 2.1491 V as the temperature changes from 20 to 80 °C, in which the output voltage varies about 0.6 mV.

## 3. Preparation of the Sensitive Film

The sensitive film of the humidity sensor was titanium dioxide prepared by the sol-gel method [25,26]. The titanium dioxide was prepared as follows: titanium isopropoxide (Ti(OC_3_H_7_)_4_) precursor of 3 mL (Henan Tianfu Chemical Co., Henan, China) was dissolved in isopropanol (C_3_H_8_O, 20 mL) (Rhett Chemical Co., Taipei, Taiwan) with stirring for 30 min until the solution was mixed uniformly. Deionized water (40 mL) was added into the mixed solution with stirring for 1 h. Then, HNO_3_ solution of 5 mL (Kaomu Co., Kaohsiung, Taiwan) was added into the mixed solution and stirred for 20 min, followed by aging for 30 min. The slurry of titanium dioxide was filtered, and then rinsed with deionized water. Finally, the titanium dioxide was coated on the substrate with calcinations at 400 °C for 1 h.

**Figure 4.**
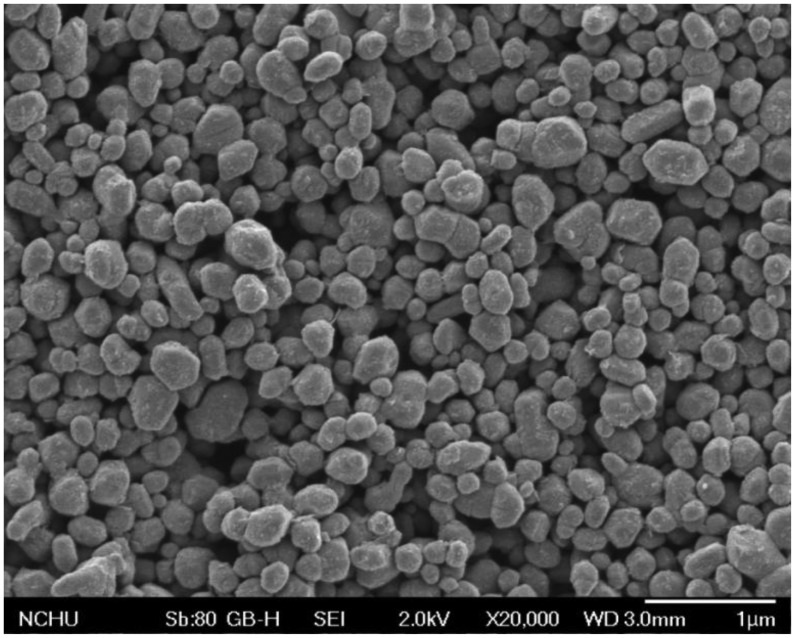
SEM image of the titanium dioxide film.

**Figure 5.**
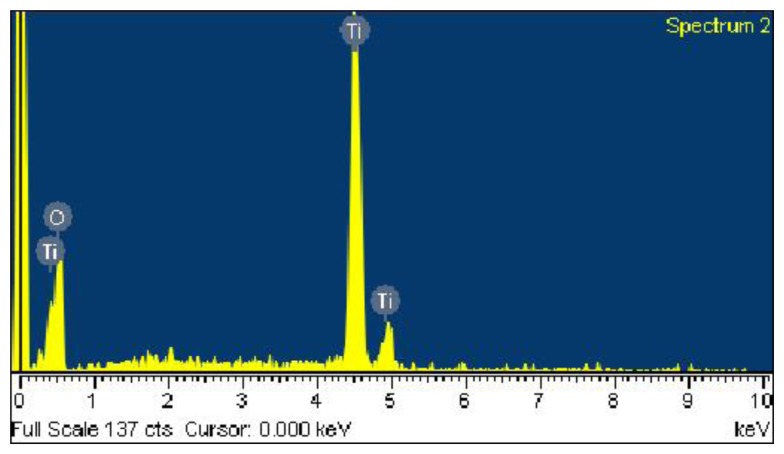
EDS analysis of the titanium dioxide film.

Scanning electron microscopy (SEM, JEOL JSM-6700F, Tokyo, Japan) was used to measure the surface morphology of the titanium dioxide film. Figure 4 presents a SEM image of titanium dioxide film. The film was nanoparticle structures that have a large surface area and helps to enhance its sensitivity. The energy dispersive spectrometer (EDS) was employed to test the composition of the titanium dioxide film. Figure 5 shows an EDS analysis of the titanium dioxide, where the main elements are titanium and oxygen. The results showed that the titanium dioxide film contained titanium of 50.75 wt% and oxygen of 49.25 wt%.

## 4. Fabrication of the Humidity Sensor

**Figure 6.**
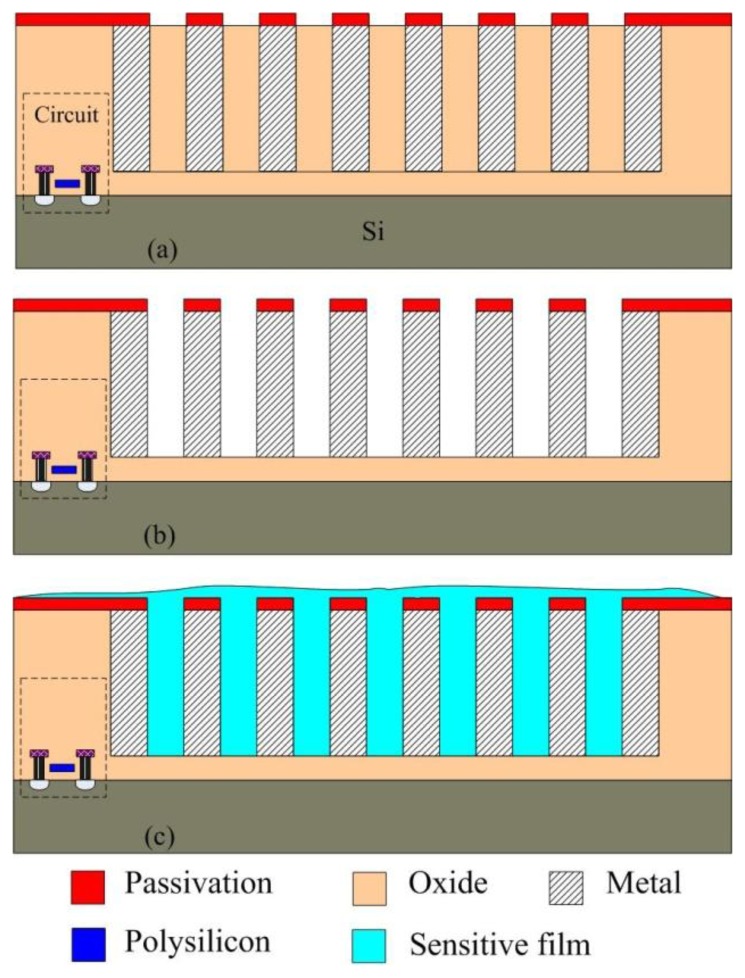
Fabrication process of the integrated humidity sensor: (**a**) after the CMOS process; (**b**) etching the sacrificial layer; (**c**) coating the sensitive film.

The commercial 0.18 μm CMOS process of Taiwan Semiconductor Manufacturing Company (TSMC, Taipei, Taiwan) was employed to manufacture the integrated humidity sensor chip. Figure 6 shows the fabrication flow of the integrated humidity sensor. The humidity sensor needed a post-process [27] to coat the sensitive film after completion of the CMOS process. The post-process contained two main steps. One etched the sacrificial layer to expose the interdigitated electrodes, and the other coated the titanium dioxide film on the interdigitated electrodes.

**Figure 7.**
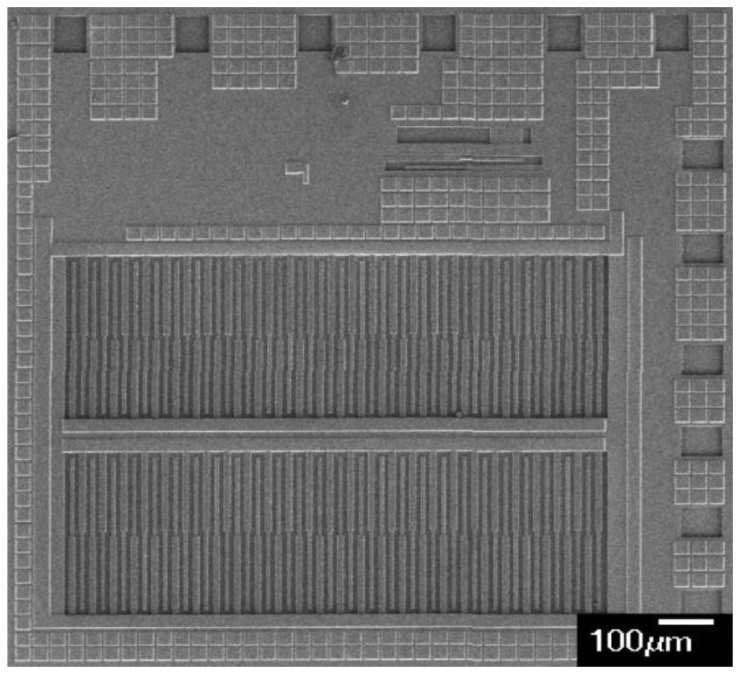
SEM images of the integrated humidity sensor after the wet etching.

**Figure 8.**
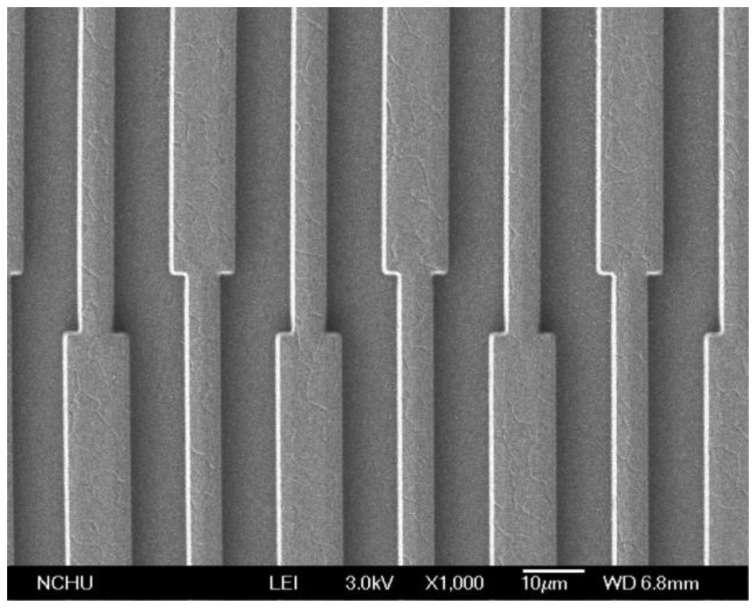
SEM image of the interdigitated electrodes.

**Figure 9.**
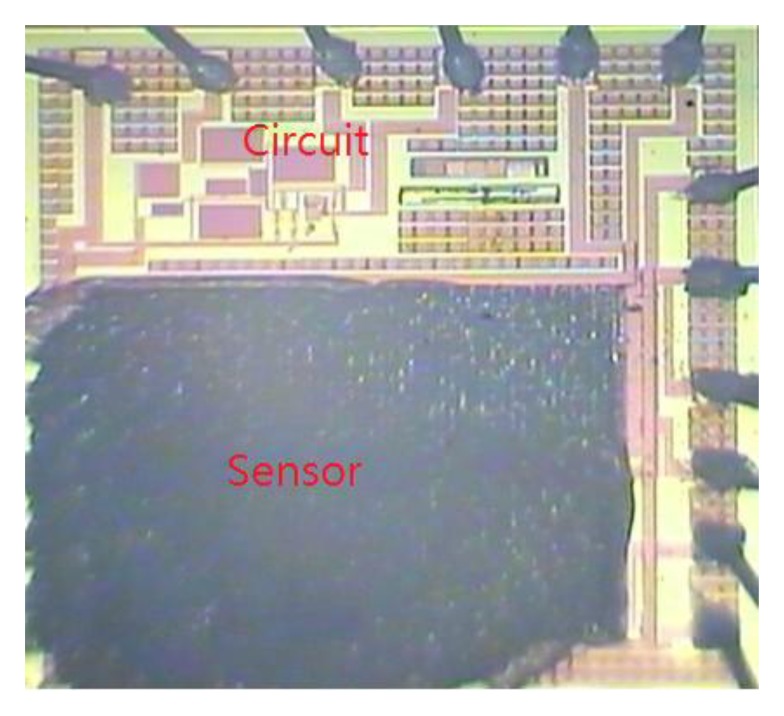
Optical image of the integrated humidity sensor after the post-process.

Figure 6a shows the cross-sectional view of the integrated humidity sensor after completion of the CMOS process. The interdigitated electrodes were made by the metal layers. Silicon dioxide located between the interdigitated electrodes was the sacrificial layer in which it needed to be removed. Figure 6b shows that the sacrificial oxide layer was removed. The sacrificial oxide layer was etched by a wet etching with buffer oxide etch (BOE) etchant [28,29], to expose the interdigitated electrodes. Figure 7 shows a SEM image of the integrated humidity sensor after the wet etching. The metal of the interdigitated electrodes was an aluminum alloy. The melting temperature of the metal was about 450 °C. Figure 8 shows a SEM image of the interdigitated electrodes after the wet etching. The interdigitated electrodes were fabricated completely. Figure 6c shows that the sensitive film was coated. A precision-control micro-dropper was used to drop the titanium dioxide onto the interdigitated electrodes, and then the titanium dioxide film was calcinated at 400 °C for 1 h. Figure 9 presents an optical image of the integrated humidity sensor after the post-process.

## 5. Results and Discussion

The performances of the humidity microsensor were tested by a power supply, a test chamber (GTH-099-40-1P, Giant Force Instruments Enterprise Co., New Taipei, Taiwan), a LCR meter and an oscilloscope. The test chamber could supply a humidity range of 25–90 RH% and a temperature range of 0–100 °C. Temperature and humidity in the test chamber could be tuned separately and maintained at constant levels. The power supply was used to provide the bias voltage and the input voltage to the readout circuit.

**Figure 10.**
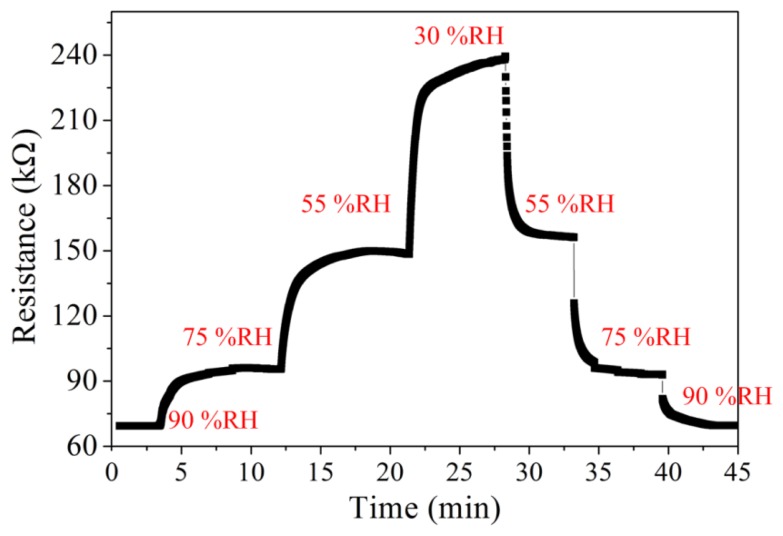
Resistance variation of the humidity sensor without readout circuit at 25 °C.

The humidity sensor without readout circuit was tested in order to characterize the resistance variation of the sensor. The humidity sensor without readout circuit was set in the test chamber, and its resistance variation under different humidity was measured by the LCR meter. Figure 10 shows the resistance variation of the humidity sensor under different humidity conditions. The measured results revealed that the resistance of the humidity sensor changed from 72 to 240 kΩ as the humidity varied from 90 to 30 RH%. The humidity sensor had a response time of 58 s and a recovery time of 65 s.

**Figure 11.**
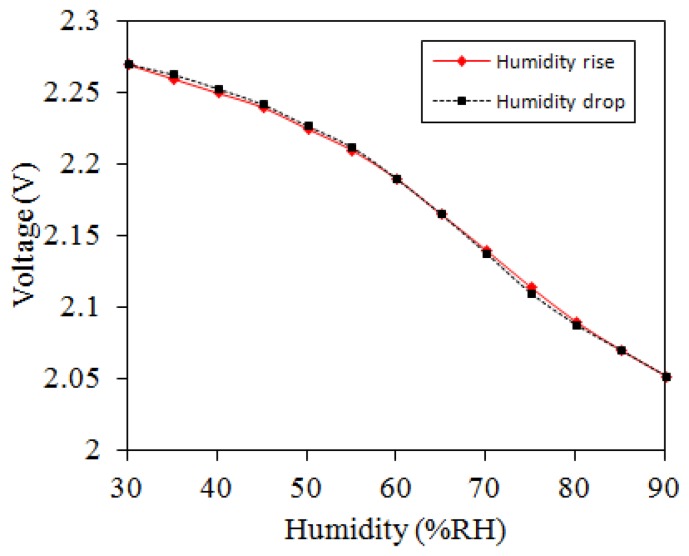
Measured results of the humidity sensor at 25 °C.

The humidity sensor with readout circuit was set in the test chamber. The test chamber provided different humidity levels to the humidity sensor. When the humidity in the test chamber rose or dropped, the resistance of the humidity sensor produced a change. The readout circuit converted the resistance variation of the sensor into the output voltage, and the oscilloscope recorded the output voltage. Figure 11 shows the measured results of the humidity sensor. In this measurement, the temperature maintained constant at 25 °C and the humidity varied from 30 to 90 RH% in 40 min and then dehumidified to 30 RH% at the same rate. The measured results showed that the output voltage of the humidity sensor changed from 2.27 to 2.05 V as the humidity increased from 30 to 90 RH%.

**Figure 12.**
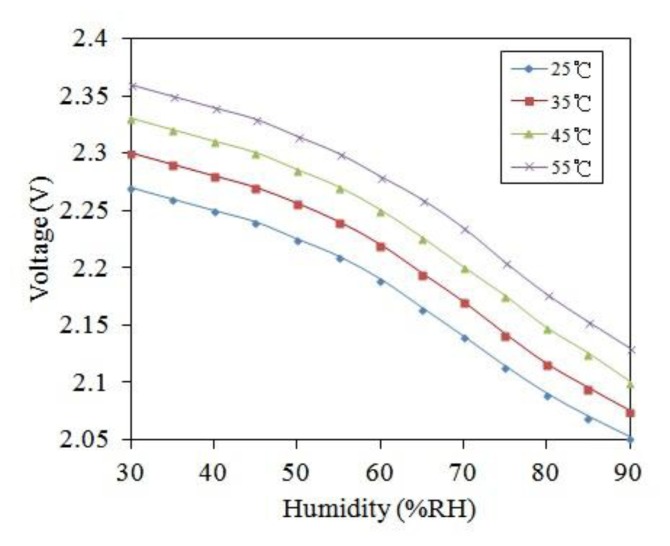
Measured results of the humidity sensor at different temperatures.

**Figure 13.**
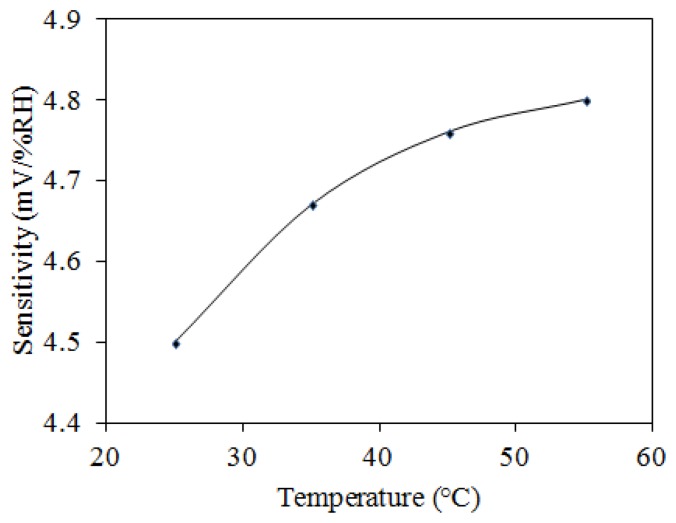
Relationship between sensitivity and temperature for the humidity sensor.

To characterize the influence of temperature, the humidity sensor was measured under different temperatures. The output voltage of the humidity sensor was detected at different temperatures. Figure 12 shows the measured results of output voltage for the humidity sensor at different temperatures. The output voltage of the humidity sensor changed from 2.27 to 2.05 V at the temperature of 25 °C as the humidity increased from 30 to 90 RH%. The output voltage of the sensor varied from 2.36 to 2.14 V at 55 °C as the humidity increased from 30 to 90 RH%. As shown in Figure 12, the curves approach linearity in the range of 45–90 RH%. According to the data in Figure 12, the relationship between the sensitivity and temperature for the humidity sensor can be obtained. Figure 13 shows the relationship between sensitivity and temperature for the humidity sensor. The results showed that the humidity sensor had a sensitivity of 4.5 mV/RH% at 25 °C and a sensitivity of 4.8 mV/RH% at 55 °C. The sensitivity of the sensor increased as the temperature increased. As shown in Figure 13, the curve is a nonlinear. The ratio of sensitivity to temperature was 0.18 mV/RH%/°C in the temperature range of 25–35 °C, and the ratio of sensitivity to temperature was 0.024 mV/RH%/°C in the temperature range of 35–55 °C.

## 6. Conclusions

A humidity sensor equipped with a readout circuit was successfully manufactured using the commercial 0.18 μm CMOS process and a post-process. The post-process was compatible with the commercial CMOS process. The humidity sensor consisted of a sensitive film and interdigitated electrodes. The sensitive film of the humidity sensor was titanium dioxide that was prepared by the sol-gel method. The titanium dioxide was deposited on the interdigitated electrodes. The humidity sensor generated a change in resistance as the sensitive film absorbed or desorbed water vapor. The resistance variation of the sensor was converted by the readout circuit into the output voltage. The sensor integrated with readout circuit on-a-chip would reduce noise and interference. The experiments showed that the output voltage of the humidity sensor decreased from 2.27 to 2.05 V as the humidity changed from 30 to 90 RH% at 25 °C. The humidity sensor had a sensitivity of 4.5 mV/RH% at room temperature.
